# Role of Radiomics in Parotid Malignant Disease: A Scoping Review

**DOI:** 10.3390/cancers17203284

**Published:** 2025-10-10

**Authors:** Andrea Migliorelli, Marianna Manuelli, Andrea Ciorba, Francesco Stomeo, Stefano Pelucchi, Chiara Bianchini

**Affiliations:** ENT & Audiology Unit, Department of Neurosciences, University Hospital of Ferrara, 44100 Ferrara, Italy

**Keywords:** parotid cancer, salivary gland cancer, artificial intelligence, radiomics, machine learning, deep learning

## Abstract

Malignant tumors of the salivary glands are rare, accounting for approximately 1–7% of all head and neck tumors. The parotid gland is the most commonly affected gland. An accurate preoperative diagnosis distinguishing between malignant and benign tumors is necessary for the appropriate management of patients. The aim of this review is to analyze the results of the most recent literature studying the use of radiomics in malignant tumors of the parotid gland. A comprehensive literature review was performed using the PubMed/MEDLINE, EMBASE and Cochrane Library databases, in accordance with the PRISMA review criteria (from 2020 to July 2025). The final analysis comprised a total of six articles and 560 patients. Four studies evaluated the role of Magnetic Resonance Imaging (MRI), one of Computed Tomography (CT) and one of Positron Emission Tomography/Computed Tomography (PET/CT). Radiomics models achieved good overall diagnostic performance, with AUC values ranging from 0.769 to 0.952 across studies, although methodological heterogeneity prevented data pooling. The results of this review indicate that radiomics has the potential to play an important role in the management of malignant tumors of the parotid gland. Nevertheless, the absence of clear and standardized protocols does not allow the comparison of results. Further studies are necessary to confirm these findings.

## Simple Summary

This review elucidates how radiomics is transforming the management of malignant neoplasms of the parotid gland: it explores the potential application of radiomics in distinguishing between benign and malignant parotid gland pathology. Radiomics is primarily based on magnetic resonance imaging. Whilst the results are encouraging, further validation is required before its adoption in clinical practice.

## 1. Introduction

The incidence of malignant tumors of the salivary glands is estimated to be between 0.5 and 2 cases per 100,000 people, accounting for around 0.3% of all tumors, 1–7% of tumors of the head and neck, and 20% of salivary tumors [1]. The parotid gland is the most common site for salivary neoplasms, though only a quarter of these are malignant. The prognosis for patients with the disease varies according to its histological type, grade and stage [2,3].

In 2017, the World Health Organization officially recognized 24 malignant and 11 benign histotypes of salivary gland tumors [4]. Although the main treatment is surgery, the extent of the procedure and the pre- and post-operative management is linked to the tumor’s histological features.

Ultrasound and magnetic resonance imaging (MRI) are recommended for the initial evaluation of a salivary neoplasm, and fine needle aspiration cytology (FNAC) is essential for determining the nature of the lesion [5,6,7,8].

At the 39th European Congress of Cytology in Milan in 2015, the ‘Milan system’ was introduced for reporting salivary gland cytopathology. This system, which is based on the estimation of the risk of malignancy, provides a shared reference for diagnosis and management and is divided into six categories [9]. Adoption of the Milan system has improved risk stratification in cytopathology and clinical patient management. However, both cytology and standard imaging methods are supportive tools, even if they are not always sufficient to guide the optimal therapeutic choices or provide reliable prognostic information [10].

Therefore, given the importance of an accurate preoperative diagnosis for proper patient management, numerous studies have sought to distinguish between benign and malignant pathologies using methods other than those traditionally employed [11,12]. In 2010, radiomics emerged in radiodiagnostics, enabling various statistics to be calculated from an image area and patterns to be extracted and not previously assessable. Furthermore, the development of artificial intelligence (AI) in medicine has certainly represented a positive turning point in radiology [13,14,15]. Consequently, numerous studies started to use radiomics and AI in the context of benign and malignant head and neck disorders [16,17,18].

The aim of this review is to analyze the results of recent literature investigating the use of radiomics in the management of parotid malignant disease.

## 2. Materials and Methods

**Figure 1:**
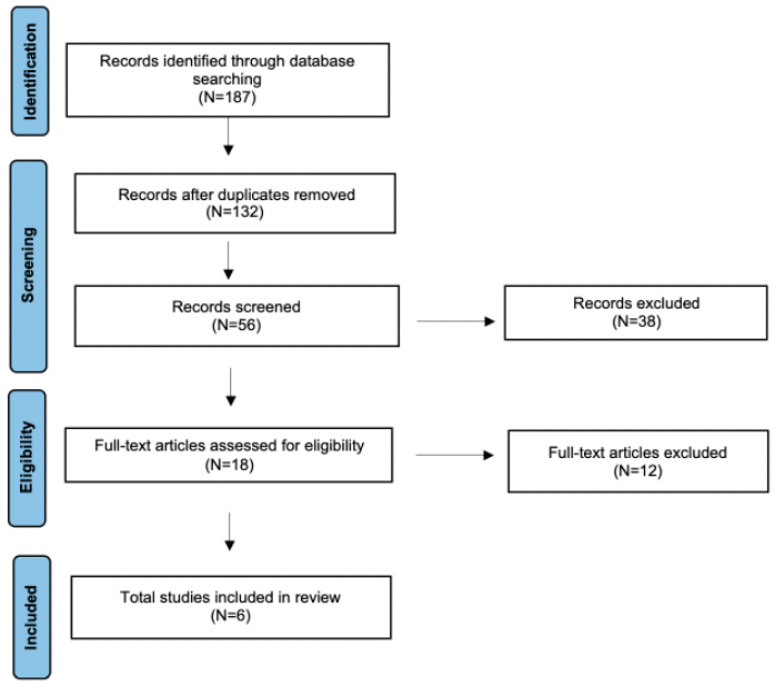
The literature review performed using PRISMA guidelines for scoping review.

A detailed review of the English-language literature on radiomics in parotid malignant tumor was performed using PubMed/MEDLINE, EMBASE, and Cochrane Library databases. The search period was from 2020 to July 2025, with the aim of selecting the most recent studies. The terms used were “parotid cancer”, “parotid tumor”, “PGT”, “MPGT” or “PGD” and “Deep Learning”, “DL”, “Machine Learning”, “ML” or “Radiomics”. The search yielded 187 candidate articles. The search was performed according to the “Preferred Reporting Items for Systematic Reviews and Meta-Analyses” (PRISMA) for scoping review guidelines (Figure 1) [19]. The inclusion criteria applied were: (i) publication date after 2020; (ii) studies aimed at using radiomics in the management of malignant tumors of the parotid gland; and (iii) English language. Conference abstracts, case reports, publications written in a language different from English have been excluded. We also excluded studies that analyzed the salivary glands in general, as well as those that evaluated the use of radiomics for distinguishing between different benign parotid tumors. Two authors (AM and MM) have evaluated independently all titles, and relevant articles have been individuated according to inclusion/exclusion criteria; a senior author (AC) resolved any disagreements. Risk of bias and applicability were assessed using the QUADAS-2 tool, which covers four domains: patient selection, index test, reference standard, and flow and timing [20]. At the end of the full-text review, 6 articles met the inclusion criteria [21,22,23,24,25,26]. The protocol for this review has been registered on the Open Science Framework (OSF) Registries with the Registration: osf.io/bxh2s. A completed PRISMA checklist is provided as a Supplementary File.

## 3. Results

This scoping review has included 6 articles, for a total of 560 patients [21,22,23,24,25,26]. The studies analyzed and their major findings are summarized in Table 1 and Table 2. The articles analyzed originate from Asia and Europe. The present review demonstrates that radiomics is predominantly employed in the management of malignant tumors of the parotid gland in combination with magnetic resonance imaging. However, two articles are also based on other imaging methods, one using CT (computed tomography) without contrast and the other using PET/CT (positron emission tomography/computed tomography). Across the included studies, the reported AUC values ranged from 0.769 to 0.952, reflecting overall good diagnostic performance. However, substantial methodological heterogeneity—including differences in imaging modality, segmentation approach, feature extraction, and validation strategy—precluded any quantitative pooling or formal meta-analysis. The QUADAS-2 assessment revealed low applicability concerns but variable risk of bias, mainly related to retrospective design, patient selection, and heterogeneous MRI protocols (Table 3).

### 3.1. MRI and Radiomics

Magnetic resonance imaging was the most used imaging modality in the studies analyzed, with four out of six papers, three of which were published in 2021; three of the four papers have been compiled in Europe.

An initial Italian study evaluated the hypothesis that magnetic resonance-based texture analysis improves diagnostic performance for the diagnosis of malignant tumors of the parotid gland [21]. The authors of the study considered a total of 57 patients who underwent 1.5 T magnetic resonance imaging. Segmentation was performed in four MRI datasets in axial images, on pre- and post-contrast axial turbo spin echo T1 sequences, SPIR (spectral presaturation with inversion recovery) sequences, and T2-weighted (T2-w) turbo spin echo sequences. The performance of radiomics was then compared with that of head and neck radiologists and non-specialist radiologists. The authors reported that for non-specialist radiologists, sensitivity, specificity and AUC (area under the curve) were 75%, 97% and 0.860; for specialist radiologists, they were 100%, 94% and 0.970. Utilizing the radiomic MRI model, which was derived from the aggregation of different sequences, the outcomes were determined to be 57.2%, 93.4%, and 0.927, respectively. Consequently, the authors conclude that radiologists with a specialized background perform better evaluations, but that radiomics can greatly assist radiologists without a specialized background [21].

In the same year, Piludu et al. [22] evaluated the role of MRI-based radiomic analysis using both T2-w images and ADC (apparent diffusion coefficient) maps in the differentiation of parotid lesions. The authors assessed 69 parotid lesions on MRI prior to surgery, and the trained model was then applied to a group of 44 patients from another center. The patients underwent 1.5 T MRI, and the radiomic analysis was based on T2 sequences and ADC maps. In the training cohort, the model for discriminating between Warthin tumors and malignant tumors achieved an accuracy of 86.7% (sensitivity 87.5%, specificity 84.6%), while in the validation group it achieved an accuracy of 77.8% (sensitivity 90%, specificity 42.9%). When evaluating the accuracy, sensitivity and specificity of the model in discriminating between benign and malignant tumors in the training and validation groups, the values reached 80.4%, 84.4% and 75% vs. 89.2%, 85% and 94.1%, respectively [22].

Consequently, in 2021, the objective of Zheng et al. [24] was to develop a radiomic MRI nomogram that would also consider clinical factors, aiming to differentiate between malignant and benign tumors of the parotid gland. The authors considered a total of 80 patients in the training group and 35 patients in the validation group. For each MRI sequence, eight groups were evaluated, resulting in the extraction of 1702 radiomic features. Subsequently, using the LASSO (Least Absolute Shrinkage and Selection Operator) algorithm, the results were further assessed obtaining 17 radiomic features, configuring the radiomic signature. Thereafter, the authors proceeded to develop a nomogram incorporating the radiomic signature with clinical data, thereby obtaining an AUC of 0.952 in the training group and 0.938 in the validation group [24].

The most recent work analyzing MRI-based radiomics was published in 2024 by Ammari et al. [25], with the aim of developing a radiomic algorithm to distinguish between different histotypes of the parotid gland. The training group comprised 86 patients, while the validation group contained 25 patients. Both 1.5 T and 3 T MRIs were performed with standard pre- and post-contrast T1-w, T2-w, and diffusion-weighted imaging (DWI) sequences. The use of ADC was considered unnecessary in comparison to DWI. Subsequently, a random forest and a logistic regression model were trained using radiomics. In the validation group, the random forest model demonstrated the most optimal outcomes, attaining an accuracy of 72%, a specificity of 86%, and a sensitivity of 72% across all histopathological subtypes. The mean AUC value was determined to be 0.838. In the discrimination between malignant and benign cases alone, an accuracy of 76% and an AUC of 0.769 were obtained. Furthermore, the authors demonstrated the efficacy of radiomics in facilitating enhanced diagnostic capabilities for radiologists not specialized in head and neck imaging [25].

### 3.2. CT-PET/CT and Radiomics

Two studies evaluated the use of radiomics based on two methods that are not MRI; both studies were performed in Asia.

The first study was conducted in 2021. In this study, the authors investigated the potential of radiomics, as determined by non-contrast CT imaging, to differentiate between benign lymphoepithelial lesions (BLEL) and mucosa-associated lymphoid tissue (MALToma) lymphoma in cases of parotid disease [23].

A radiomic nomogram was developed that comprehended both clinical factors and radiomic signatures. Following the conclusion of LASSO regression, a total of seven features were identified as contributing to the radiomic signature. The authors demonstrated that the radiomic nomogram has an AUC of 0.983 and 0.950 in the training and validation groups, respectively, in differentiating between BLEL and MALToma [23].

Furthermore, in 2025, Nakajo et al. [26] utilized an advanced machine learning (ML) and deep learning (DL) approach, incorporating radiomic features of [18F]-FDG-PET/CT, to achieve a precise differentiation between benign and malignant parotid tumors. The training group comprised 44 patients, while the validation group consisted of 19 patients. A total of 49 radiomic features were used to differentiate between benign and malignant tumors of the parotid gland. Five distinct ML algorithms and DL-based tests were used for the analysis. The latter achieved optimal results with an AUC of 1.000 in the training group (100% accuracy) and an AUC of 0.976 in the test group (95% accuracy) [26].

## 4. Discussion

This scoping review analyses the role of radiomics in managing malignant tumors of the parotid gland. The analyzed studies come from Europe and Asia. Four of the studies were based on MRI [21,22,24,25], one on CT [23] and one on PET/CT [26]. Regarding the use of MRI to distinguish between benign and malignant tumors, we found that the AUC ranged from 0.952 to 0.769, with an accuracy ranging from 89.2% to 76% [21,22,24,25].

MRI is currently one of the most widely used imaging techniques for studying the parotid gland, particularly neoplasms, due to its ability to provide detailed characterization of soft tissues. Numerous studies have demonstrated that MRI’s diagnostic performance is often comparable to FNAC, which remains the gold standard for preoperative characterization of salivary lesions [27,28].

However, the introduction of radiomics, in recent years, has opened up new perspectives in the study of medical images [13]. This term refers to the process of converting digital images into a large set of quantitative data, which is extracted using mathematical algorithms that can describe the characteristics of a lesion in an objective and reproducible way. In head and neck oncology, radiomics has produced promising results in terms of diagnosis, patient management and follow-up [13]. Therefore, it is not surprising that radiomics has also been applied with growing interest to the study of the parotid gland using MRI, with several studies analyzing its potential for characterizing neoplasms [21,22,23,24,25,26].

Our review shows that MRI-based radiomics produces extremely favourable results, with most studies achieving an AUC value greater than 0.80, and often even 0.90. These results suggest that the method has considerable potential for distinguishing between benign and malignant tumors or identifying specific histological subtypes. Furthermore, the most interesting data appear to be obtained from T2-wsequences [21,22,24,25].

Additionally, the most significant results were achieved using the radiomic nomogram, which integrates the radiomic signature with clinical factors, yielding an AUC of 0.952 [24]. The increasing interest in radiomics is also associated with the shift towards personalized and precision medicine. The information that radiomics can provide goes beyond the morphology that is visible to the radiologist; the quantitative parameters extracted reflect tissue properties related to cellular, metabolic, and even genomic processes that would otherwise be inaccessible through direct observation [29]. This approach expands the role of imaging, from a purely descriptive tool to a true non-invasive biomarker.

Nevertheless, methodological differences between studies remain an obstacle to its implementation in everyday clinical practice, limiting its application to the scientific field [29,30]. Radiomics is characterized by a complex workflow involving several stages, from image acquisition and lesion segmentation to data pre-processing, feature extraction and selection, and modelling. Each step can introduce significant variability in the results. Currently, there is no international consensus on standardized protocols, which limits the reproducibility and generalizability of the proposed models [31].

Therefore, its application remains limited to research, due to the complexity of the workflow and the variability of the methods used, which reduces the repeatability of studies.

A recent systematic review on the use of MRI-based radiomics to assess salivary gland tumors revealed promising results in the individual studies analyzed. However, no radiomic feature has been shown to recur consistently in the final models. The absence of shared protocols has also led to heterogeneous methodological approaches being used. Several models have indeed been proposed, but without the adoption of a uniform strategy that would allow comparability and validation. These findings strongly emphasize the need to develop and implement standardized radiomic analysis procedures for defining salivary tumors using MRI [30]. Aringhieri et al. [29] reached the same conclusion in their systematic review of the methodological quality of salivary gland radiomics studies.

It should be noted that almost all of the studies analyzed used both a training and a validation sample, either internal or external. However, as reported in the literature, the absence of clear and shared protocols makes it difficult to compare the different results.

The potential of radiomics extends beyond MRI; as demonstrated by Zheng et al. [23], who used non-contrast CT to distinguish between BLEL and MALToma of the parotid gland. Using a radiomic nomogram that integrated radiomic signatures and clinical factors, the authors achieved significant results, with an AUC of 0.983 in the training group and 0.950 in the validation group. If these results are confirmed in further studies, they could represent a valuable resource for the management and diagnosis of this disease.

The increasing use of PET/CT in oncology, and its inclusion in major international guidelines, has recently led to this method being widely studied in the head and neck area [32,33]. Consequently, PET/CT was also employed for differential diagnosis between benign and malignant tumors in 2025. Integrating radiomic results with ML and DL methods achieved impressive results, with an accuracy of between 100% and 95% in the training and validation groups, respectively [26].

In the literature, radiomics has been applied to the salivary glands, extending beyond the neoplastic aspect. For example, it has been used to study post-radiotherapy xerostomia, which is one of the most common toxicities experienced by patients undergoing treatment for head and neck tumors. Several studies have evaluated the possibility of using MRI or CT image analysis to predict which patients are at higher risk of developing this complication early on, with the aim of personalizing treatment planning and improving long-term quality of life [34,35]. Radiomics is also emerging as a tool for diagnosing inflammatory diseases such as sialadenitis and Sjögren’s syndrome [36,37]. Although evidence is still limited, some studies have proposed radiomics-based scores to improve diagnostic accuracy in complex scenarios.

However, a major limitation to the clinical translation of radiomics is the absence of standardized acquisition and analysis protocols. Differences in imaging parameters, segmentation methods, and feature extraction can lead to variability in results and limit reproducibility across centers. The establishment of harmonized workflows, multicenter collaborations, and the use of shared datasets are essential steps to improve consistency, validate predictive models, and enable the reliable application of radiomics in clinical practice.

Another key finding of the present review is the role of radiomics and AI in supporting radiologists and residents who are not specializing in head and neck imaging in evaluating parotid lesions. Numerous studies reported that the results of radiomic models are superior to those of radiologists without expertise in this field, but inferior to those with expertise [21,25]. This is particularly important for rare diseases such as malignant tumors of the parotid gland. Radiomics could therefore be a valuable tool for radiologists, even in centers where these diseases are uncommon.

In the future, the development of radiomic methods alongside the increasing use of ML and DL will be essential for achieving ever greater diagnostic accuracy and providing patients with increasingly personalized treatment. Furthermore, improving outcomes could also be achieved by implementing MRI alongside the wider availability of 3 T technology.

Future research should aim to address current methodological gaps, particularly through multicenter collaborations ensuring standardized MRI acquisition and radiomic feature extraction across different platforms (1.5 T and 3 T). Studies including external validation cohorts and cost-effectiveness assessments compared with conventional diagnostic approaches such as FNAC would further support the clinical applicability of radiomics in parotid gland tumors.

Main drawbacks of this study are: (i) the limited number of studies currently available in the literature, resulting in a limited scope for this review, (ii) retrospective studies and (iii) the heterogeneity of the radiomics models used, making comparisons difficult.

## 5. Conclusions

Magnetic resonance-based radiomics represents a valuable emerging approach for the characterization of parotid gland malignant tumors, showing encouraging diagnostic performance in recent studies. However, the methodological heterogeneity and the lack of standardized protocols currently limit its clinical application, and the comparability of the research studies available. In selected scenarios, CT and PET/CT can also offer a valuable contribution, expanding the potential applications of radiomics in the study of malignant tumors of the parotid gland. In our opinion, as this method becomes more widespread and integrated with artificial intelligence tools, it will be important to develop shared, validated procedures in order to allow its clinical applicability, particularly in order to achieve tailored patient management.

Future research should prioritize prospective multicenter studies with standardized imaging and analysis protocols to improve reproducibility and validation. External testing and integration of radiomics with clinical and pathological data will be essential to strengthen model generalizability. Furthermore, prospective studies assessing clinical utility and cost-effectiveness are needed to facilitate the translation of radiomics into routine management of parotid gland malignancies.

## Figures and Tables

**Table 1:** Literature review.

Author (Yrs)	Country	Modality	Reference Standard	N Training/N Validation	Segmentation	Software	ICC/Repeatability	Validation Type	AUC (95% CI)	Calibration/Clinical Utility
Vernuccio (2021) [21]	Italy	MRI	H	0/57	Manual ROI (MRI T1, T2, post-contrast)	Horos/MATLAB	ICC variable (poor–excellent)	Internal (cross-validation)	Best radiomics benign vs. malignant: AUC 0.927; head & neck radiologist AUC 0.970 (95% CI 0.934–1.000)	NA
Piludu (2021) [22]	Italy	MRI	H	69/44	Lesion delineation	S-IBEX/3D Slicer	ICC used for feature selection	External (independent cohort)	Accuracy 86.7–91.9%; AUC not explicitly stated	Calibration curve + DCA
Zheng (2021) [23]	China	CT	H	70/31	Semi-automatic	NA	ICC ≥ 0.75	External (two-center)	Nomogram AUC 0.952 (train), 0.938 (val)	DCA performed; good calibration
Zheng (2021) [24]	China	MRI	H	80/35	Manual	NA	ICC > 0.80	External (split-sample)	Radiomics + clinical nomogram AUC 0.983 (train), 0.950 (val)	Good calibration; DCA positive net benefit
Ammari (2024) [25]	France	MRI	H	86/25	Manual	Olea Sphere 3.0.x	NA	Internal (train/test)	Random Forest multi-class accuracy 0.720; OvR AUC 0.838; benign/malignant AUC 0.769	NA
Nakajo (2025) [26]	Japan	PET/CT	H	44/19	VOI	LIFEx v6.00	NA	Internal (train/test)	Ensemble DL model AUC 0.976 (test)	NA

Legend: Yrs: years, N: numbers, MRI: magnetic resonance imaging, CT: computed tomography, PET: positron emission tomography, H: Histopathology, NA: not available, ICC = Intraclass Correlation Coefficient, DCA = Decision Curve Analysis, DL: deep learning.

**Table 2:** Objective and Major Results.

Author (Yrs)	Objective	Major Results
Vernuccio (2021) [21]	Evaluate whether MRI-based texture analysis improves the differentiation of malignant parotid tumors and distinguishes pleomorphic adenoma from Warthin tumor compared with conventional imaging.	Radiologists showed higher accuracy than radiomics in detecting malignancy, while a T2-weighted texture-based model improved non-specialists’ differentiation of pleomorphic adenoma and Warthin tumor.
Piludu (2021) [22]	Assess MRI-based radiomics using T2-weighted images and ADC maps to differentiate parotid lesions, and validate predictive models on an external cohort.	Radiomic analysis of ADC and T2-weighted images, combined with qualitative features such as margins and contrast enhancement, achieved good-to-excellent diagnostic accuracy in differentiating parotid lesions.
Zheng (2021) [23]	Develop and externally validate a CT-based radiomics-plus-clinical nomogram to preoperatively differentiate parotid BLEL from MALT lymphoma.	The radiomic nomogram integrating clinical factors and radiomic features effectively differentiated parotid BLEL from MALT lymphoma, achieving high predictive performance (AUC = 0.983 for training and 0.950 for validation).
Zheng (2021) [24]	Develop and validate a radiomic nomogram based on magnetic resonance imaging to differentiate between benign and malignant parotid gland tumors preoperatively.	Incorporating clinical factors and the radiomic signature, the radiomic nomogram achieved an AUC value of 0.952 in the training set and 0.938 in the validation set.
Ammari (2024) [25]	Present a machine learning algorithm that can classify parotid gland tumors into their respective histopathological subtypes.	The random forest model performed best, achieving a multi-class accuracy of 0.720 and an average OvR AUC of 0.838 on the test set. It achieved OvR accuracies of 0.840 for pleomorphic adenomas and Warthin tumors, and 0.760 for carcinomas (benign/malignant distinction). All features selected by the algorithm originated from the T2w sequence.
Nakajo (2025) [26]	Use radiomic features to develop and identify ML models based on PET/CT in order to differentiate between benign and malignant parotid gland diseases.	In the test group, three of the five conventional ML models achieved an AUC greater than 0.80 (range: 0.815–0.929). The DL-based ensemble model achieved the highest AUC value (0.976).

Legend: Yrs. Years, MRI: magnetic resonance imaging, CT: computed tomography, PET: positron emission tomography, w: weight, ML: machine learning, DL: deep learning, AUC: area under the curve (AUC = 1.0: perfect discrimination; AUC = 0.5: no discriminative power), ADC: apparent diffusion coefficient, BLEL: benign lymphoepithelial lesion, MALT: mucosa-associated lymphoid tissue, OvR: One-vs-Rest.

**Table 3:** QUADAS-2 assessment.

Author (Yrs)	Patient Selection	Index Test	Reference Standard	Flow & Timing	Applicability Concerns
Vernuccio (2021) [21]	High	Low	Unclear	High	Low–Moderate
Piludu (2021) [22]	High	Unclear	Low	Unclear	Low
Zheng (2021) [23]	Low	Low	Low	Low	Low
Zheng (2021) [24]	Low	Low	Low	Low	Low
Ammari (2024) [25]	High	Low	Low	Unclear	Moderate
Nakajo (2025) [26]	Unclear	Low	Unclear	Unclear	Low–Moderate

Legend: Yrs. Years.

## Supplementary Materials

The following supporting information can be downloaded at: https://www.mdpi.com/article/10.3390/cancers17203284/s1. PRISMA 2020 checklist.
